# Toward evidence-based neuroarchitecture: a systematic mapping and framework for immersive spatial cognition

**DOI:** 10.3389/fpsyg.2026.1919278

**Published:** 2026-09-07

**Authors:** Ezgi Yılmaz, Mehmet Sair Akkam, Fatma Sedes

**Affiliations:** 1 Faculty of Architecture, Istanbul Aydin University, Istanbul, Türkiye; 2 Faculty of Engineering and Architecture, İstanbul Gelişim University, Center for Environment, Urban Studies and Geology, Istanbul, Türkiye

**Keywords:** electroencephalography, environmental psychology, evidence-based design, Evidence-Based Neuroarchitecture Maturity Model, immersive environments, Immersive Spatial Cognition Framework, neuroarchitecture, spatial cognition

## Abstract

Neuroarchitecture has developed rapidly at the intersection of architecture, environmental cognition, neuroscience, and immersive technologies, yet the field remains methodologically heterogeneous and its progression from measurement toward design application is insufficiently structured. This study addresses this gap through a PRISMA 2020-compliant systematic mapping review of neuroarchitecture and closely related architectural and spatial-cognition research published between 2015 and 2025. Expanded searches across Scopus and Web of Science Core Collection identified 510 records. After removal of 206 duplicates, 304 unique records were screened; 125 records entered the final eligibility and data-extraction audit, and four additional boundary or temporal exclusions resulted in a final corpus of 121 studies. The corpus comprised 61 human empirical studies, one non-human empirical study, 58 review or conceptual synthesis records, and one methodological/protocol record. Publication activity increased markedly in the later review period, with 81 studies (66.9%) published between 2023 and 2025. Among the 61 human empirical studies, questionnaire/self-report measures were used in 39 studies (63.9%), behavioral task/performance measures in 32 (52.5%), EEG in 32 (52.5%), eye tracking in 15 (24.6%), EDA/GSR in 14 (23.0%), HRV in six (9.8%), fMRI in three (4.9%), and fNIRS in one (1.6%). To organize this heterogeneous evidence base, the study develops an Immersive Spatial Cognition Framework (ISCF) linking spatial stimuli, environmental exposure, neurophysiological measurement, cognitive–emotional processing, evidence interpretation, design translation, and adaptive design, together with an Evidence-Based Neuroarchitecture Maturity Model (EBN-MM). EBN-MM classification indicated that 30.6% of the corpus remained at Stage I (Conceptual Interpretation), 35.5% reached Stage II (Experimental Validation), 33.1% reached Stage III (Evidence Integration), and only 0.8% reached Stage IV (Adaptive Design Operationalization). These findings indicate that neuroarchitecture is progressing beyond isolated experimental validation toward evidence integration, while translation into adaptive and operational design remains exceptional. The proposed frameworks provide a structured basis for comparing heterogeneous evidence and identifying methodological priorities for future evidence-based neuroarchitecture research.

## 1 Introduction

Contemporary built environments are increasingly evaluated not only through physical performance and spatial functionality, but also through their influence on cognitive health, emotional regulation, psychological wellbeing, and human environmental experience (Sternberg and Wilson, 2006; Coburn et al., 2017). In recent years, growing concerns regarding stress-related environmental conditions, mental wellbeing, restorative spatial quality, and human-centered sustainability approaches have substantially expanded the scope of architectural research beyond conventional environmental performance criteria (Attaianese et al., 2025; Mallgrave, 2013). This transformation has contributed to the emergence of immersive spatial cognition research, where spatial environments are examined not only as physical settings, but also as cognitive-emotional systems capable of shaping neurophysiological and behavioral responses.

Within this transformation, neuroarchitecture has emerged as an interdisciplinary field integrating architecture, neuroscience, environmental psychology, and cognitive science to investigate how spatial environments affect perception, cognition, emotional adaptation, and neurophysiological activity (Coburn et al., 2017; Eberhard, 2009). Early neuroarchitectural discourse was predominantly shaped by neuroaesthetic and phenomenological interpretations emphasizing sensory perception, spatial atmosphere, emotional response, and embodied environmental experience (Mallgrave, 2013; Vartanian et al., 2013). While these studies significantly expanded theoretical discussions regarding psychologically responsive environments, much of the early literature remained conceptual and perception-oriented without measurable neurophysiological validation.

In recent years, the methodological structure of neuroarchitecture has changed considerably due to developments in immersive technologies, wearable neurophysiological systems, and digital environmental simulations (Kuliga et al., 2015; Taherysayah et al., 2024). VR-supported spatial simulations, EEG-based environmental evaluations, biometric monitoring systems, and immersive cognitive experiments increasingly enable researchers to investigate spatial experience through quantifiable neurophysiological and cognitive evidence (Shemesh et al., 2017; Banaei et al., 2017; Gramann et al., 2017). Similarly, immersive virtual environments have become increasingly important for examining cognitive-emotional responses to restorative, biophilic, and healthcare-oriented environments (Kalantari and Neo, 2020; Kim and Gero, 2023).

At the same time, restorative environmental psychology, biophilic design research, and evidence-based healthcare environments have become increasingly integrated into neuroarchitectural discourse (Ulrich et al., 2008; Browning et al., 2014; Yin et al., 2018). Nevertheless, methodological consistency across immersive environmental cognition research remains uneven. While perceptual and emotional responses are frequently foregrounded, systematic integration between immersive simulations, neurophysiological evidence systems, and sustainability-oriented spatial interpretation remains inconsistently operationalized (Taherysayah et al., 2024; Albayrak-Kutlay, 2025).

Taken together, three structural asymmetries emerge within contemporary neuroarchitecture scholarship:

(1) Rapid conceptual expansion of neuroarchitectural discourse without systematic evidence stratification.
(2) Increasing adoption of VR- and EEG-supported environmental experimentation without unified Immersive Spatial Cognition Frameworks.
(3) Growing sustainability-oriented and restorative environmental discourse without sufficiently integrated neurophysiological validation models.

To address this gap, the present study conducts a PRISMA 2020-compliant systematic mapping review of neuroarchitecture and closely related architectural and spatial-cognition research published between 2015 and 2025. Following an expanded multi-set search of Scopus and Web of Science Core Collection, 121 studies were retained for systematic mapping and evidence classification. The study proposes the Immersive Spatial Cognition Framework (ISCF) and the Evidence-Based Neuroarchitecture Maturity Model (EBN-MM) as complementary analytical tools for organizing heterogeneous evidence and examining the field’s progression from conceptual interpretation and experimental validation toward evidence integration and adaptive design operationalization.

## 2 Literature review

### 2.1 Neuroaesthetic foundations: conceptual expansion without evidence stratification

Early neuroarchitectural discourse developed primarily through neuroaesthetic theory, phenomenological interpretation, and embodied environmental perception research (Sternberg and Wilson, 2006; Mallgrave, 2013). Foundational studies emphasized the relationship between architectural space, sensory experience, emotional response, and cognitive adaptation, positioning built environments as psychologically responsive systems (Coburn et al., 2017; Chatterjee and Vartanian, 2014; Vartanian et al., 2013). In parallel, neuroarchitecture gradually expanded interdisciplinary interaction between architecture, neuroscience, environmental psychology, and cognitive science (Eberhard, 2009; Attaianese et al., 2025).

However, despite substantial conceptual growth, methodological evidence stratification across neuroarchitectural research remains limited. Existing studies continue to prioritize phenomenological interpretation, perceptual discourse, and emotional-spatial association, while measurable neurophysiological validation remains inconsistently integrated (Medhat Assem et al., 2023; de Paiva and Jedon, 2019). Although neuroarchitecture increasingly presents itself as an evidence-oriented field, much of the literature still lacks unified analytical structures capable of comparatively linking environmental cognition, immersive experimentation, and measurable neurophysiological response.

### 2.2 Immersive technologies and neurophysiological experimentation: methodological expansion without unified cognitive frameworks

Recent neuroarchitectural research increasingly incorporates immersive technologies, virtual simulations, and neurophysiological monitoring systems to investigate human-environment interaction through measurable cognitive and physiological responses. VR-supported spatial simulations have been widely adopted for examining emotional adaptation, spatial perception, environmental preference, and restorative environmental experience under controlled experimental conditions (Kuliga et al., 2015; Kalantari and Neo, 2020; Valentine, 2023). Parallel developments in EEG-based environmental evaluation further expanded the analytical capacity of immersive environmental research (Shemesh et al., 2017; Banaei et al., 2017; Gramann et al., 2017).

Nevertheless, methodological consistency across immersive environmental experimentation remains comparatively fragmented. Taherysayah et al. (2024) identify substantial variability regarding experimental protocols, EEG interpretation models, environmental simulation structures, and cognitive response evaluation methods within VR-supported architectural research. Existing studies frequently prioritize simulation-based experimentation and controlled immersive environments, while comparative integration between measurable neurophysiological evidence, environmental cognition models, and design-oriented spatial interpretation remains limited (Albayrak-Kutlay, 2025; Grobman, 2026).

### 2.3 Neurophysiological measurement and evidence integration: analytical growth without operational consistency

Recent neuroarchitectural research employs neurophysiological and biometric measurement systems to investigate cognitive, emotional, and physiological responses to architectural environments. EEG, HRV, GSR, eye-tracking systems, and emerging fNIRS-supported environmental experiments are progressively incorporated into immersive environmental cognition studies (Shemesh et al., 2017; Banaei et al., 2017; Llinares et al., 2021; Higuera-Trujillo et al., 2021). Parallel developments in wearable sensing technologies and real-time physiological monitoring have further expanded evidence-oriented environmental evaluation (Gramann et al., 2017; Edelstein and Macagno, 2012).

Nevertheless, operational consistency across neurophysiological environmental research remains comparatively fragmented. Existing studies demonstrate substantial variability regarding EEG interpretation protocols, physiological indicator selection, biometric synchronization procedures, environmental exposure conditions, and cognitive response classification methods. While physiological measurement systems increasingly provide measurable environmental evidence, translational integration between neurophysiological outputs, spatial cognition models, and architectural translation remains limited (Taherysayah et al., 2024; Rizzo et al., 2023).

### 2.4 Restorative environments and human-centered sustainability: expansion without integrated neurophysiological validation

Restorative environments, biophilic design strategies, and evidence-based healthcare settings have become central themes in human-centered sustainability research. Evidence-based healthcare design has demonstrated that environmental conditions can influence recovery processes and psychological comfort, while biophilic design research extends this discussion by emphasizing nature-related spatial qualities, daylight, materiality, and multisensory experience (Ulrich et al., 2008; Browning et al., 2014; Yin et al., 2018; Kim and Gero, 2023).

However, methodological integration across restorative environmental research remains uneven. Many studies continue to prioritize perceived restoration, emotional response, and subjective wellbeing, while measurable neurophysiological validation is less consistently embedded within assessment procedures (Bower et al., 2019; Valentine, 2023). Thus, contemporary restorative environmental research demonstrates substantial conceptual and interdisciplinary expansion, yet the integration of restorative design discourse, measurable neurophysiological evidence, and Immersive Spatial Cognition Frameworks remains methodologically fragmented.

### 2.5 Research gap and framework positioning

Across neuroaesthetic discourse, immersive environmental experimentation, neurophysiological measurement research, and restorative environmental studies, the lack of systematic cross-domain integration remains evident. Although neuroarchitectural literature has expanded considerably, these research trajectories often develop through disconnected methodological structures (Attaianese et al., 2025; Grobman, 2026; de Paiva, 2025).

Taken together, contemporary neuroarchitecture research reveals three recurring structural conditions:

(1) Fragmented integration between conceptual, immersive, physiological, and restorative research domains.

(2) Rapid methodological expansion without consistent evidence stratification.

(3) Limited architectural translation of immersive and neurophysiological environmental findings.

No existing review has mapped this transition across the full spectrum from neuroaesthetic theory to operational adaptive design, nor proposed a field-level maturity model to guide evidence accumulation. The present study addresses this gap in three ways. First, it provides a PRISMA 2020-compliant systematic mapping of an expanded 121-study evidence base spanning conceptual, empirical, review, and methodological contributions relevant to neuroarchitecture and architectural/spatial cognition. Second, it proposes the Immersive Spatial Cognition Framework (ISCF), a seven-layer analytical structure linking spatial stimuli and environmental exposure to neurophysiological measurement, cognitive-emotional processing, evidence interpretation, design translation, and adaptive design. Third, it introduces the Evidence-Based Neuroarchitecture Maturity Model (EBN-MM), which classifies the developmental position of the evidence base across four stages: Conceptual Interpretation, Experimental Validation, Evidence Integration, and Adaptive Design Operationalization. Together, these frameworks are intended to clarify not only which methods are being used, but also how far the resulting evidence has progressed toward architectural translation.

## 3 Methodology

### 3.1 Research design

The present study was designed as a systematic mapping review following the PRISMA 2020 reporting guidelines (Page et al., 2021). The review aims to examine how immersive spatial cognition research has developed across different methodological and conceptual trajectories within the built-environment field, and to identify the structural transitions characterizing the field’s movement toward evidence-based practice. Four principal research domains organized the analytical structure: (a) neuroaesthetic and perceptual environmental research; (b) VR-supported immersive environmental experimentation; (c) neurophysiological and biometric measurement studies; and (d) restorative and biophilic environmental research.

### 3.2 . Information sources, search strategy, and reporting standards

Publications were identified through systematic searches of the Scopus and Web of Science (WoS) Core Collection databases. These databases were selected because of their broad coverage of architecture, environmental psychology, neuroscience, and interdisciplinary built-environment research. The review was designed and reported in accordance with the PRISMA 2020 Statement (Page et al., 2021) and followed the principles of systematic mapping, as the primary objective was to classify the conceptual, methodological, technological, and translational development of the field rather than to estimate pooled intervention effects.

The review protocol was not prospectively registered because the study was designed as an exploratory systematic mapping review and framework-development exercise rather than an intervention-focused systematic review or meta-analysis. To support transparency and reproducibility, the database retrieval structure, screening audit, eligibility decisions, coding rules, controlled vocabulary, decision log, and analytical dataset are documented in Supplementary materials S1–S7.

Following concerns that a search restricted to the literal terms “neuroarchitecture” and “neuro-architecture” would underrepresent the broader evidence base relevant to architectural cognition, the search strategy was expanded through complementary search sets. The expanded conceptual scope encompassed neuroarchitecture and neuro-architecture; architectural, spatial, and environmental cognition; cognitive mapping, spatial memory, spatial perception, navigation, and wayfinding; built-environment research employing neurophysiological, psychophysiological, behavioral, or oculometric measures; and immersive or virtual architectural environments. Related restorative, biophilic, healthcare, educational, workplace, residential, and urban studies were considered only when the built environment and cognitive, behavioral, or neurophysiological response constituted a central component of the study.

Searches were implemented using the TITLE-ABS-KEY fields in Scopus and the Topic (TS) field in Web of Science Core Collection. The expanded search comprised three complementary search sets in Scopus (S1–S3) and three in Web of Science (W1–W3). Scopus yielded 221, 4, and 10 records across S1–S3, respectively, producing a database subtotal of 235 records. Web of Science yielded 220, 10, and 45 records across W1–W3, respectively, producing a subtotal of 275 records. The combined database retrieval therefore comprised 510 records.

Searches were restricted to English-language peer-reviewed journal articles and reviews published between 2015 and 2025. Conference proceedings, editorials, letters, book chapters, dissertations, and other non-peer-reviewed publication types were excluded. For records first published online within the review period but subsequently assigned to a later issue, temporal eligibility was evaluated according to the documented publication-status rule in the coding decision log.

The complete search architecture, database-specific search-set identifiers, retrieval counts, restrictions, and reproducibility notes are provided in Supplementary material S1. The archived project records preserve the retrieval structure and counts for all six expanded search sets; however, the exact line-by-line Boolean syntax was not retained for every expanded set. Consequently, Boolean expressions that could not be verified from the archived search history were not retrospectively reconstructed.

### 3.3 Study selection and screening process

Study selection followed the PRISMA 2020 workflow and was managed in Rayyan (Ouzzani et al., 2016). Records retrieved from the six database search sets were combined before duplicate removal and screening. Screening decisions were evaluated against the predefined scope of the review, which required a substantive relationship between the built environment or architectural/spatial characteristics and cognitive, behavioral, experiential, or neurophysiological responses.

Stage 1—Identification. The expanded searches identified 510 records, comprising 235 records from Scopus and 275 from Web of Science Core Collection.

Stage 2—Duplicate removal. After database outputs were combined, 206 duplicate records were removed, leaving 304 unique records for screening. The duplicate count was determined from the audited difference between the complete retrieval set and the unique screening set (510–304 = 206); potential-duplicate groups generated by screening software were not treated as equivalent to the number of records removed.

Stage 3—Screening and Quality-Control Review. The 304 unique records were evaluated against the predefined eligibility criteria. Studies were retained when architecture, spatial configuration, environmental exposure, or built-environment characteristics constituted a substantive component of the research question and were examined in relation to cognition, perception, affect, behavior, navigation, neurophysiology, psychophysiology, or closely related response domains. Records were excluded when the built environment was peripheral to the research question, when the study addressed general health or wellbeing without a relevant spatial or architectural mechanism, or when the publication fell outside the specified publication-type criteria. Screening and subsequent quality-control review excluded 179 records, leaving 125 records for the final eligibility and data-extraction audit.

Stage 4—Final Eligibility and Data-Extraction Audit. All 125 provisionally retained records were entered into the study-level Master Dataset and subjected to a final eligibility and coding audit. This stage identified four additional records that did not satisfy the finalized scope or temporal criteria. One clinical mindfulness intervention lacked a relevant architectural or built-environment exposure; one urban wellbeing survey did not directly examine architectural cognition, navigation, or a neurophysiological mechanism; one broad biophilic-design review treated neuroarchitecture only peripherally; and one publication had a 2026 version-of-record date and therefore fell outside the predefined 2015–2025 review period. Following these four exclusions, the final systematic mapping corpus comprised 121 studies.

The final corpus included 61 human empirical studies, one non-human empirical study, 58 review or conceptual-synthesis records, and one methodological/protocol record. These evidence strata were retained because the objectives of the mapping review extend beyond estimating empirical effects to examining the conceptual, methodological, and translational development of neuroarchitecture. They were nevertheless separated analytically: review-level or conceptual references to physiological techniques were not counted as original empirical measurements, protocol-level planned measurements were excluded from observed empirical frequencies, and the non-human study was excluded from human-participant denominators.

**Figure 1:**
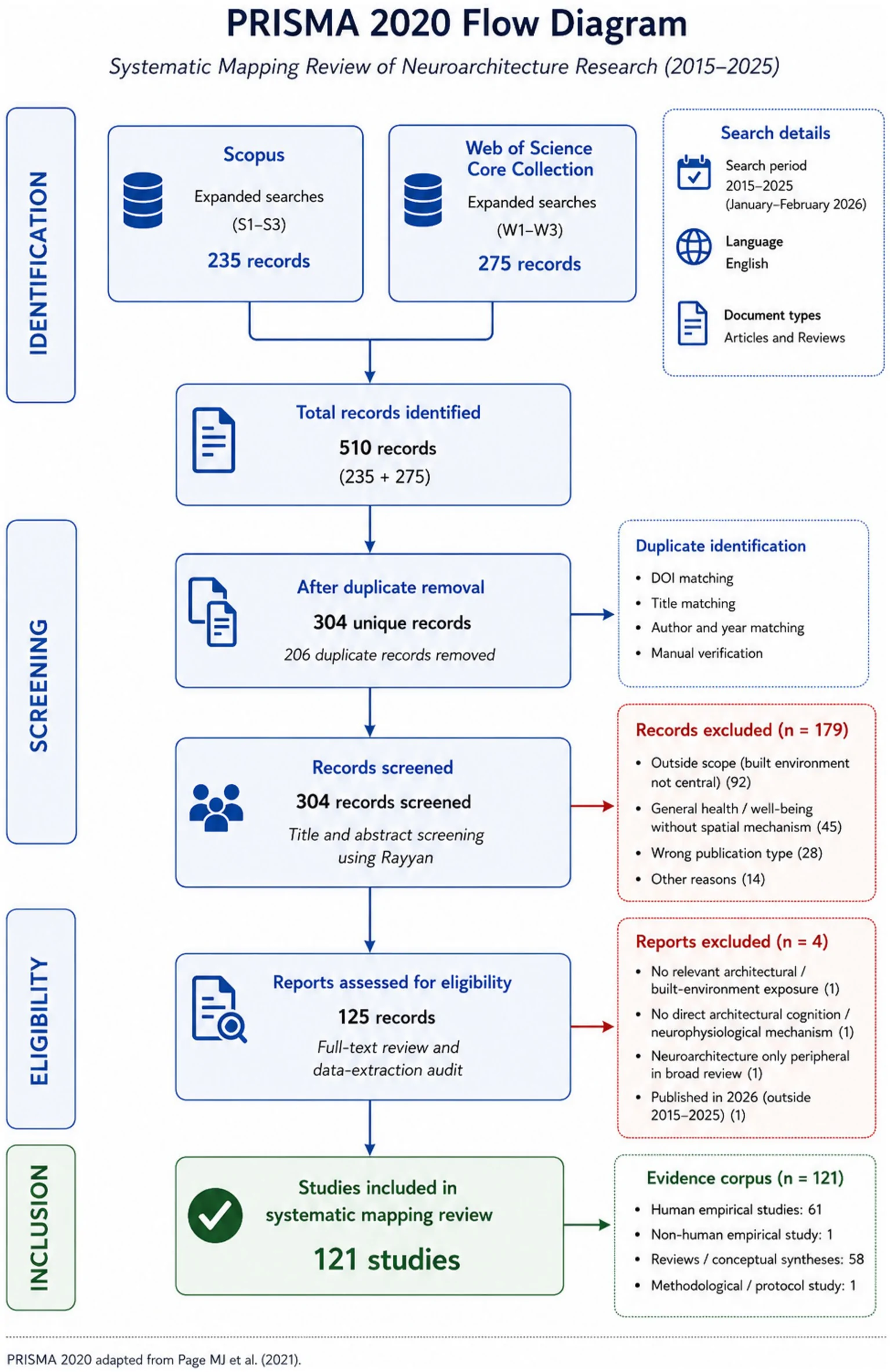
PRISMA 2020 flow diagram for the expanded systematic mapping review. Searches of Scopus and Web of Science Core Collection identified 510 records. After removal of 206 duplicates, 304 unique records were screened; 179 records were excluded during screening and quality-control review, leaving 125 records for the final eligibility and data-extraction audit. Four additional records were excluded following the final eligibility audit, resulting in 121 studies included in the systematic mapping corpus.

The complete PRISMA audit trail is provided in Supplementary material S2, the final flow counts and expanded-search log in S3, and the characteristics of all 121 retained studies in S4. Figure 1 summarizes the selection process.

### 3.4 Data extraction and coding framework

A structured study-level data extraction and coding framework was developed to enable systematic comparison across the 121 retained publications. Each publication constituted one analytical case and retained a unique Study_ID throughout the Master Dataset, supplementary files, analytical matrices, and framework classifications. Multiple coding was permitted when a study explicitly reported more than one exposure technology, measurement modality, physiological metric, architectural variable, cognitive or emotional construct, or behavioral outcome.

Coding followed an explicit-reporting rule. Variables were assigned only when supported by the title, abstract, keywords, or verified full text. Information was not inferred solely from disciplinary convention or from the presence of another variable. Thus, specific EEG frequency bands were not inferred from the use of EEG; HRV indices were not inferred from generic cardiac monitoring; cognitive or emotional constructs were not inferred directly from physiological signals; and specific architectural variables were not inferred from a generic environmental context. Where the accessible source supported only a broader level of description, coding was retained at that level rather than being imputed.

Six principal analytical domains were coded: (1) exposure technologies, (2) measurement modalities, (3) physiological metrics, (4) architectural variables and environmental contexts, (5) cognitive and emotional constructs, and (6) behavioral outcomes. Exposure technology was treated independently from measurement modality. For example, immersive virtual reality describes how an architectural environment was presented, whereas EEG, eye tracking, HRV/ECG, EDA/GSR, heart rate, fMRI, and fNIRS describe how participant responses were measured.

Publication type was additionally coded to preserve appropriate analytical denominators. Original human empirical studies were eligible for applicable empirical modality and metric analyses. Review, conceptual, and framework publications were retained for evidence synthesis but excluded from counts of original physiological observations. The non-human empirical study was retained as a distinct evidence stratum but excluded from human-participant denominators. Protocol or methodological records describing planned measurements were retained for methodological synthesis without being counted as observed empirical measurements.

Two framework-oriented classifications were subsequently applied to the retained corpus. The Evidence-Based Neuroarchitecture Maturity Model (EBN-MM) classified studies into four levels: Stage I, Conceptual Interpretation; Stage II, Experimental Validation; Stage III, Evidence Integration; and Stage IV, Adaptive Design Operationalization. To prevent maturity inflation, the use of machine learning for signal cleaning, prediction, or classification alone was not considered sufficient for Stage IV. Stage IV required demonstrated integration of user-state or neurophysiological evidence into an adaptive, generative, or operational design workflow.

The Immersive Spatial Cognition Framework (ISCF) organized the evidence across seven primary layers: L1, Spatial Stimulus/Conceptual Foundation; L2, Immersive Environmental Exposure; L3, Neurophysiological Measurement; L4, Cognitive–Emotional Processing; L5, Evidence-Based Environmental Interpretation; L6, Evidence-Based Design Translation; and L7, Adaptive/AI-Supported Design. A primary layer was assigned to each study according to its principal analytical contribution, while the underlying study-level variables remained available for cross-domain analyses.

The final coding architecture and audit trail are distributed across the Supplementary materials S4 provides the characteristics of the 121 retained studies; S5 contains the frozen study-level Master Dataset; S6A provides the coding manual; S6B defines the controlled vocabulary; S6C documents exceptional and boundary coding decisions; and S7 provides the final analytical dataset. All final tables, figures, descriptive statistics, EBN-MM classifications, and ISCF mappings were derived from these synchronized data sources.

## 4 Results

The following sections present the descriptive and framework-based findings derived from the final corpus of 121 retained studies. Because the corpus includes original empirical studies as well as review, conceptual, and methodological contributions, denominators are reported explicitly for each analysis. Frequencies of observed measurement modalities are calculated from the 61 human empirical studies, whereas publication trends, study-type distributions, EBN-MM stages, and ISCF primary-layer classifications refer to the complete 121-study corpus.

### 4.1 Publication trends (2015–2025)

The temporal distribution of the 121 retained studies indicates substantial growth in research relevant to neuroarchitecture and architectural/spatial cognition during the latter part of the review period. Twenty studies (16.5%) were published between 2015 and 2020, a further 20 (16.5%) appeared in 2021–2022, and 81 studies (66.9%) were published between 2023 and 2025.

Annual publication counts further demonstrate this acceleration. The retained corpus included three studies published in 2016, three in 2017, two in 2018, five in 2019, seven in 2020, ten in 2021, ten in 2022, 17 in 2023, 25 in 2024, and 39 in 2025. Thus, approximately two-thirds of the mapped literature was published during the final three years of the review period.

**Figure 2:**
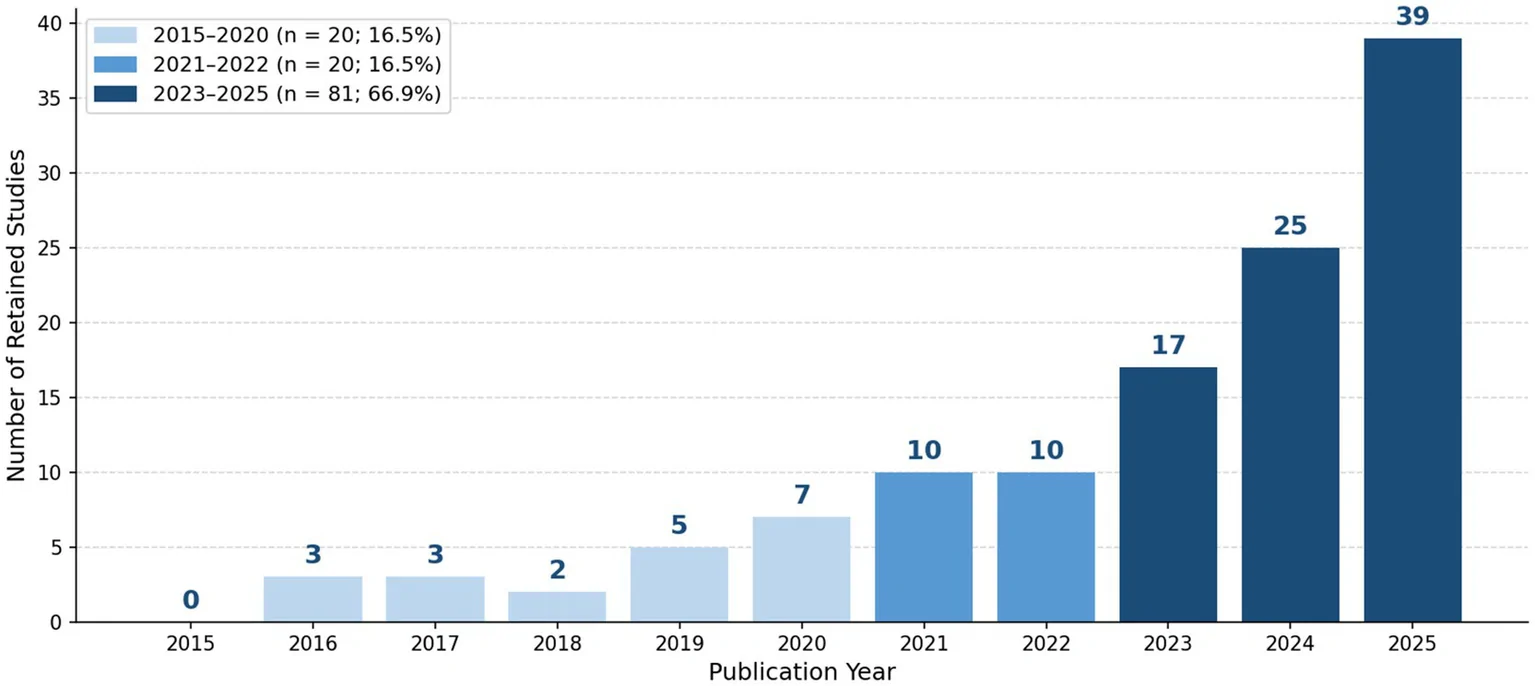
Annual publication frequency of the retained systematic mapping corpus (*n* = 121) between 2015 and 2025. The three analytical periods comprise 2015–2020 (*n* = 20; 16.5%), 2021–2022 (*n* = 20; 16.5%), and 2023–2025 (*n* = 81; 66.9%). (Source: Prepared by the authors).

This pattern indicates rapid recent expansion of the evidence base. Importantly, however, publication growth should not be interpreted by itself as evidence of methodological maturity. The subsequent analyses therefore distinguish publication volume from study design, empirical measurement, evidence integration, and design translation (Figure 2).

### 4.2 Methodological profile of the evidence base

The expanded corpus demonstrates substantial methodological diversity. Of the 121 retained studies, 34 were experimental empirical studies, 22 employed mixed-method empirical designs, five were qualitative empirical studies, and one was observational empirical research. The synthesis-oriented literature comprised 18 systematic reviews, 15 narrative reviews, 12 conceptual or theoretical contributions, two scoping reviews, and one bibliometric review. In addition, 10 publications were classified as methodological or framework contributions and one as a protocol.

For analytical purposes, these publication types were consolidated into evidence strata. Sixty-one studies constituted the human empirical evidence base, one provided non-human empirical evidence, 58 contributed at the review or conceptual-synthesis level, and one protocol record was retained as methodological evidence without being counted as observed empirical measurement.

The near balance between original human empirical research (61/121; 50.4%) and synthesis/conceptual contributions (58/121; 47.9%) indicates that the field currently combines active experimental development with substantial efforts to consolidate, conceptualize, and structure its emerging knowledge base. This composition also reinforces the need to distinguish observed empirical measurements from techniques or findings discussed only at review or conceptual level.

### 4.3 Measurement and evidence profile

Measurement frequencies were evaluated separately within the 61 human empirical studies to avoid treating measurement techniques mentioned in reviews or conceptual publications as original observations. Questionnaire or self-report measures were identified in 39 studies (63.9%), while behavioral task or performance measures were used in 32 (52.5%). EEG was also employed in 32 studies (52.5%), making it the most frequent directly coded neurophysiological modality in the human empirical corpus.

Other physiological and oculometric techniques were less frequent. Eye tracking was identified in 15 studies (24.6%), EDA/GSR in 14 (23.0%), HRV in six (9.8%), fMRI in three (4.9%), and fNIRS in one (1.6%). Because studies could employ more than one measurement modality, these percentages are non-mutually exclusive and therefore do not sum to 100%.

The resulting profile indicates that contemporary neuroarchitecture cannot be characterized as a predominantly EEG-based field alone. Rather, its empirical structure combines subjective reporting, behavioral performance, neurophysiological measurement, and oculometric or autonomic indicators. This multimethod structure creates opportunities for evidence triangulation while simultaneously increasing the need for transparent reporting and modality-specific standardization.

### 4.4 Neurophysiological and behavioral measurement trends

Within the human empirical evidence base (*n* = 61), questionnaire/self-report assessment represented the most frequently coded response method (*n* = 39; 63.9%). EEG and behavioral task/performance measures were each identified in 32 studies (52.5%). Eye tracking (*n* = 15; 24.6%) and EDA/GSR (*n* = 14; 23.0%) formed a second group of comparatively established measurement approaches, whereas HRV (*n* = 6; 9.8%), fMRI (*n* = 3; 4.9%), and fNIRS (*n* = 1; 1.6%) remained less frequently represented.

These frequencies demonstrate that the empirical evidence base extends beyond direct neural recording. Behavioral and self-reported outcomes remain central components of neuroarchitectural investigation and frequently coexist with physiological measures. Accordingly, neurophysiological evidence should be interpreted as one component of a broader multimethod architecture–human response research structure rather than as a substitute for behavioral or experiential assessment.

**Figure 3:**
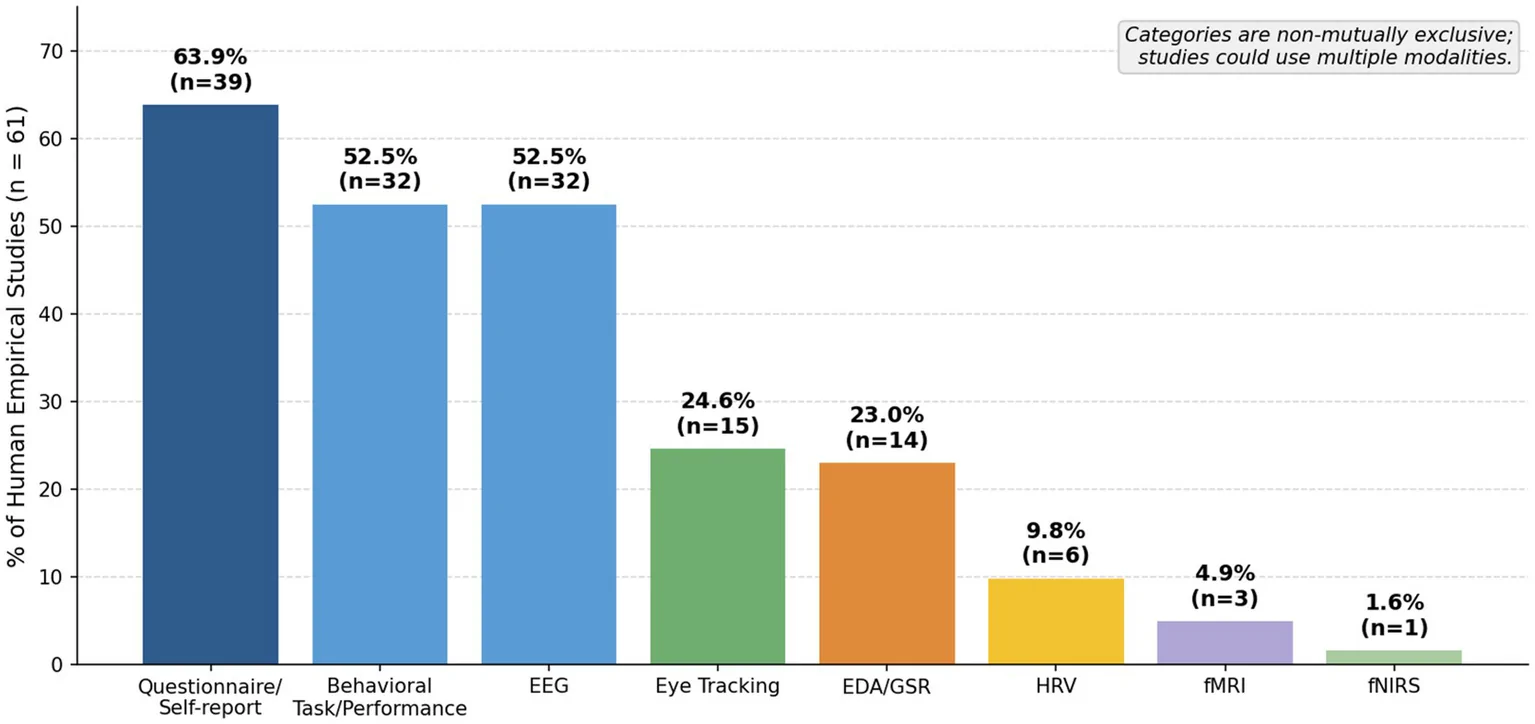
Frequency of selected measurement modalities among human empirical studies (*n* = 61). Categories are non-mutually exclusive because individual studies could employ multiple measurement modalities. (Source: prepared by the authors).

The coding audit also revealed substantial heterogeneity in the level of physiological reporting. Specific signals or metrics were retained only where explicitly reported or verified; EEG frequency bands, HRV indices, and related metrics were not inferred from generic modality labels. Consequently, the reported frequencies represent conservative study-level evidence rather than reconstructed methodological assumptions (Figure 3).

A notable limitation across EEG-based studies was the substantial variability in electrode configurations, frequency band selection, artifact rejection protocols, and cognitive interpretation models, consistent with Taherysayah et al.’s (2024) finding of fragmented EEG methodology across 19 VR + EEG architectural studies.

### 4.5 Evidence progression across the EBN-MM

Application of the Evidence-Based Neuroarchitecture Maturity Model to the complete corpus revealed a comparatively balanced distribution across the first three developmental stages. Stage I—Conceptual Interpretation comprised 37 studies (30.6%), Stage II—Experimental Validation comprised 43 studies (35.5%), and Stage III—Evidence Integration comprised 40 studies (33.1%). In contrast, only one study (0.8%) met the conservative criteria for Stage IV—Adaptive Design Operationalization.

This distribution modifies the interpretation suggested by the earlier, narrower evidence base. The expanded corpus is not dominated by experimental validation alone. Instead, conceptual interpretation, experimental validation, and evidence integration each represent approximately one-third of the literature. The principal maturity discontinuity therefore occurs between evidence integration and operational design translation.

Stage IV classification was deliberately conservative. The presence of machine-learning analysis, automated classification, or computational prediction was not sufficient by itself to indicate adaptive design operationalization. Stage IV required demonstrated integration of user-state or neurophysiological evidence into an adaptive, generative, or operational design workflow. Under this criterion, only one retained study reached the final maturity stage.

### 4.6 Distribution across the Immersive Spatial Cognition Framework

Primary-layer classification within the ISCF showed that 47 of the 121 studies (38.8%) were positioned at L3—Neurophysiological Measurement, representing the largest single layer in the corpus. L1—Spatial Stimulus/Conceptual Foundation included 27 studies (22.3%), while L5—Evidence-Based Environmental Interpretation included 21 (17.4%). L4—Cognitive–Emotional Processing accounted for 13 studies (10.7%), and L6—Evidence-Based Design Translation for 11 (9.1%). Only one study (0.8%) was assigned primarily to L2—Immersive Environmental Exposure and one (0.8%) to L7—Adaptive/AI-Supported Design.

The distribution indicates a pronounced concentration around measurement and interpretation. Neurophysiological measurement is comparatively well represented, and a substantial synthesis-oriented literature has emerged at L5. By contrast, relatively few studies occupy the design-translation layer, and empirically demonstrated adaptive or AI-Supported Design remains exceptional.

This asymmetry is central to the ISCF interpretation: the field has developed considerable capacity to expose participants to architectural conditions, measure responses, and interpret resulting evidence, but the downstream conversion of that evidence into operational architectural decision systems remains comparatively underdeveloped.

### 4.7 EBN-MM and ISCF integration

To synthesize the methodological evolution identified throughout the mapping process, the retained studies were conceptualized within a four-stage developmental model. The proposed Evidence-Based Neuroarchitecture Maturity Model (EBN-MM) is presented in Table 1.

**Table 1:** Evidence-Based Neuroarchitecture Maturity Model (EBN-MM) and distribution of the final systematic mapping corpus (*n* = 121).

Stage	Label	Operational interpretation	Corpus distribution
Stage I	Conceptual Interpretation	Conceptual, theoretical, narrative, or framework-level interpretation without original empirical validation or formal systematic synthesis	37 (30.6%)
Stage II	Experimental Validation	Original empirical investigation using behavioral, physiological, qualitative, or mixed-method assessment; methodological validation without adaptive implementation	43 (35.5%)
Stage III	Evidence Integration	Formal evidence synthesis, multimodal physiological integration, or advanced analytical integration without demonstrated adaptive design operation	40 (33.1%)
Stage IV	Adaptive Design Operationalization	Demonstrated integration of user-state/neurophysiological evidence into an adaptive, generative, or operational design workflow	1 (0.8%)

Taken together, the EBN-MM and ISCF results reveal a consistent structural pattern. The field has established substantial conceptual, experimental, and evidence-integration capacity, but this accumulated evidence rarely progresses to adaptive design operationalization. The EBN-MM therefore represents maturity in terms of the degree of evidence translation rather than a simple hierarchy of methodological sophistication.

Correspondingly, the ISCF should not be interpreted as implying that every study must individually traverse all seven layers. Instead, the framework represents a field-level analytical chain through which heterogeneous studies can be positioned according to their primary contribution. The empirical distribution across the seven layers demonstrates where evidence is currently concentrated and where translational discontinuities remain.

**Figure 4:**
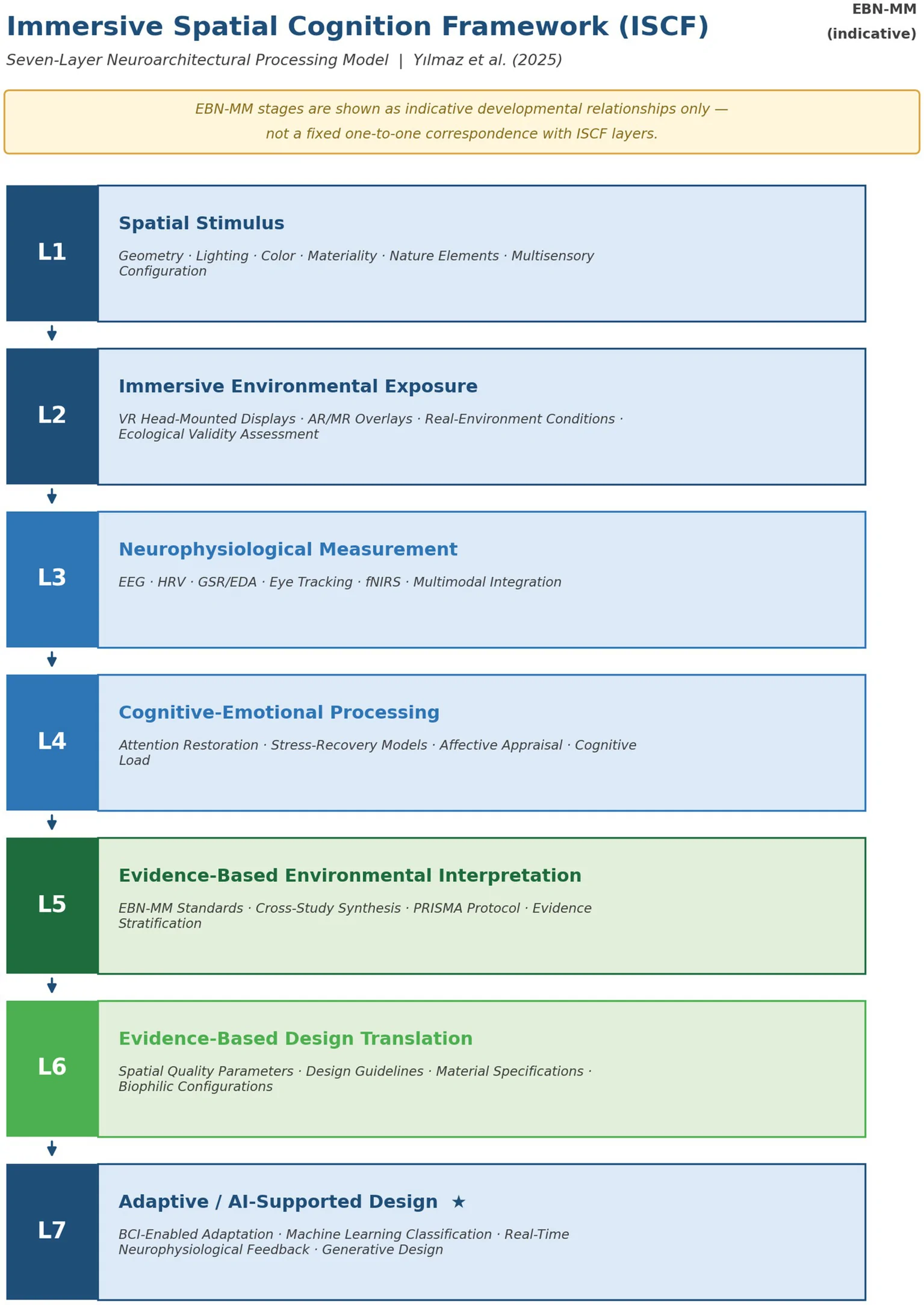
ISCF seven-layer processing model from spatial stimulus (layer 1) to adaptive/AI-supported design (layer 7). (Source: Prepared by the authors).

Building upon the identified methodological trajectory, the proposed ISCF integrates the sequential processes linking spatial stimuli to adaptive evidence-based design. Figure 4 illustrates the complete seven-layer framework.

## 5 The Immersive Spatial Cognition Framework (ISCF)

Building upon the systematic mapping findings, the ISCF provides a unified seven-layer analytical model for neuroarchitectural research and practice. The framework integrates the four primary research domains identified in the literature review within a sequential processing chain from spatial stimulus input to AI-supported adaptive design output.

To facilitate practical implementation, each layer of the ISCF is operationalized through specific analytical functions and design outcomes, as summarized in Table 2.

**Table 2:** The Immersive Spatial Cognition Framework (ISCF): seven-layer structure from spatial stimulus to adaptive design.

Layer	Label	Description
L1	Spatial Stimulus	Physical and virtual architectural conditions: geometry, lighting, color, materiality, nature elements, multisensory configurations
L2	Immersive Environmental Exposure	Controlled delivery of spatial stimuli via VR HMDs, AR overlays, or real-environment conditions; ecological validity assessment
L3	Neurophysiological Measurement	Multimodal data acquisition: EEG (cognitive load, attention, arousal), HRV (autonomic regulation), GSR (emotional activation), eye tracking (attentional allocation), fNIRS (prefrontal engagement)
L4	Cognitive–Emotional Processing	Integration of physiological signals within validated cognitive-emotional models: attention restoration theory, stress-recovery, affective appraisal frameworks
L5	Evidence-Based Environmental Interpretation	Translation of cognitive-emotional outputs into evidence-graded spatial cognition findings; EBN-MM evidence standards; cross-study synthesis
L6	Evidence-Based Design Translation	Conversion of synthesized findings into design-actionable guidelines: spatial quality parameters, material specifications, biophilic configurations
L7	Adaptive/AI-Supported Design	Real-time integration of neurophysiological user-state data with AI-driven design systems; BCI-enabled spatial adaptation; generative design optimization

Together, Figure 4 and Table 2 establish both the conceptual architecture and the operational structure of the proposed Immersive Spatial Cognition Framework (ISCF). While Figure 4 illustrates the sequential relationships among the seven analytical layers, Table 2 operationalizes each layer by defining its principal functions and expected design outcomes. Collectively, these complementary representations provide a reproducible framework that can support future empirical neuroarchitecture research, facilitate cross-study comparisons, and guide the translation of neurophysiological evidence into evidence-based architectural design practice.

**Figure 5:**
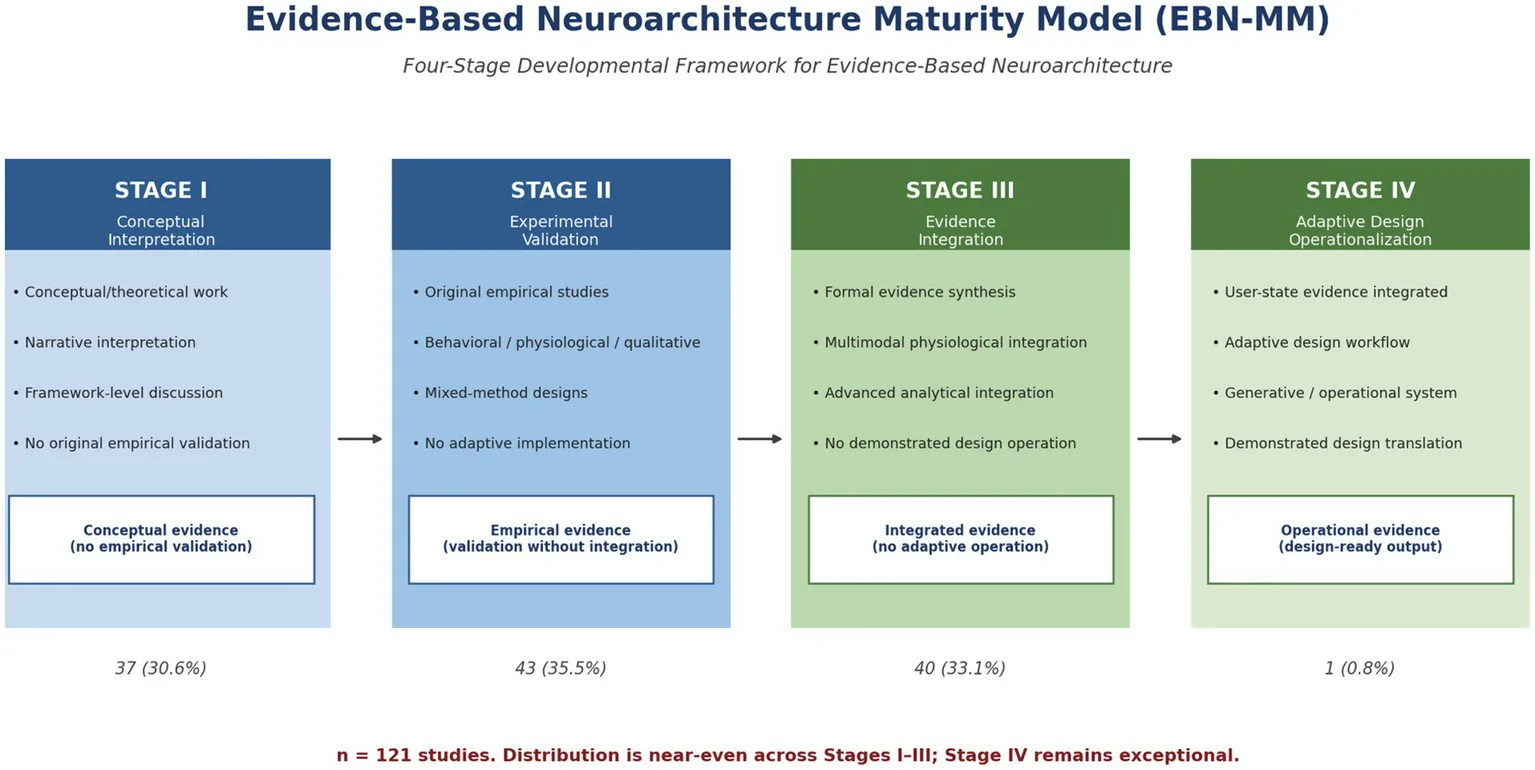
EBN-MM distribution across the final systematic mapping corpus (*n* = 121): Stage I, 37 studies (30.6%); Stage II, 43 (35.5%); Stage III, 40 (33.1%); and Stage IV, one (0.8%). (Source: Prepared by the authors).

As illustrated in Figure 5, the current literature is distributed in comparable proportions across Stages I, II, and III, indicating substantial conceptual, experimental, and evidence-integration activity while simultaneously revealing the limited transition toward fully adaptive, AI-supported neuroarchitectural applications.

A distinctive contribution of the ISCF relative to existing frameworks—including Taherysayah et al.’s (2024) VR-EEG review framework, Grobman’s (2026) SPEC model, and Attaianese et al.’s (2025) built-environment framework—is its explicit inclusion of Layer 7, which positions AI-supported adaptive design as the terminal stage of the proposed neuroarchitectural processing chain. The corpus analysis supports the broader translational gap identified by the framework: only one of the 121 retained studies (0.8%) reached Stage IV of the EBN-MM, while only one study (0.8%) was primarily classified at L7—Adaptive/AI-Supported Design. Together, these findings indicate that adaptive and operational design applications remain emergent rather than established within the field.

The study-level ISCF and EBN-MM classifications are provided in the Supplementary materials rather than reproduced as a 121-row table in the main manuscript. These classifications were treated as complementary analytical dimensions rather than as a fixed one-to-one correspondence between ISCF layers and EBN-MM stages. As reported in Sections 4.5 and 4.6, the corpus is concentrated at L3—Neurophysiological Measurement, while the EBN-MM distribution remains comparatively balanced across Stages I–III. The operational classification rules and study-level assignments are documented in Supplementary materials S6A–S7.

## 6 Discussion

### 6.1 Rapid growth does not equate to uniform methodological maturity

The expanded systematic mapping reveals a rapidly growing but structurally heterogeneous field. Of the 121 retained studies, 81 (66.9%) were published between 2023 and 2025, demonstrating a marked recent acceleration in research connecting architecture, spatial cognition, immersive environments, and human-response measurement. However, publication growth should not be conflated with methodological maturity. The EBN-MM distribution is almost evenly divided across Conceptual Interpretation (30.6%), Experimental Validation (35.5%), and Evidence Integration (33.1%), indicating that conceptual development, empirical experimentation, and synthesis are advancing in parallel rather than through a simple linear transition.

This finding nuances narratives that portray contemporary neuroarchitecture primarily as a shift from conceptual discourse toward physiological experimentation. Experimental research is now substantial, but conceptual and synthesis-oriented work remains equally important to the structure of the field. The principal developmental challenge is therefore not merely to increase the number of empirical studies, but to strengthen the pathways through which heterogeneous findings are consolidated and translated into architectural knowledge.

### 6.2 Neuroarchitecture is methodologically multimodal rather than technology-defined

The measurement profile further challenges a technology-centered definition of neuroarchitecture. Among the 61 human empirical studies, questionnaire/self-report measures were identified in 63.9%, while behavioral task/performance measures and EEG were each used in 52.5%. Eye tracking and EDA/GSR occurred in approximately one-quarter of the empirical corpus, whereas HRV, fMRI, and fNIRS were substantially less frequent.

These findings suggest that neuroarchitecture is better understood as a multimethod research domain than as a field defined by any single neurophysiological technology. EEG occupies an important position, but behavioral and subjective-response methods remain equally central to understanding architectural experience. The methodological challenge is therefore one of triangulation: physiological signals acquire greater interpretive value when considered alongside behavioral performance, cognitive appraisal, and experiential reporting rather than being treated as self-sufficient indicators of architectural response.

### 6.3 The main translational gap lies beyond measurement

The ISCF distribution provides further evidence that the central bottleneck in contemporary neuroarchitecture occurs downstream of measurement. L3—Neurophysiological Measurement represented the largest primary layer, accounting for 38.8% of the corpus. Evidence synthesis and environmental interpretation at L5 were also comparatively well represented (17.4%). However, only 9.1% of studies were primarily positioned at L6—Evidence-Based Design Translation, and only one study (0.8%) reached L7—Adaptive/AI-Supported Design.

This pattern indicates that the field’s most pressing problem is no longer simply whether architectural experience can be measured. Rather, it concerns how measured and synthesized evidence can be transformed into reproducible architectural decision rules. The distinction is important because increasingly sophisticated measurement does not automatically produce evidence-based design. Translation requires explicit relationships between environmental variables, measured human responses, interpretive models, and actionable design parameters.

### 6.4 Adaptive design remains an emerging rather than established paradigm

The EBN-MM reinforces this interpretation. Only one of the 121 retained studies met the conservative Stage IV criterion. Importantly, machine learning, automated classification, or AI-assisted analysis was not considered sufficient for Stage IV unless neurophysiological or user-state evidence was demonstrably connected to an adaptive, generative, or operational design workflow.

This distinction prevents computational sophistication from being conflated with architectural translation. AI may support signal classification or prediction without altering a design decision, just as physiological monitoring may quantify response without generating an actionable spatial intervention. Stage IV therefore represents a substantially higher translational threshold: evidence must participate directly in a design-operational process.

The scarcity of Stage IV evidence should consequently be interpreted as an identified research frontier rather than as a weakness of the field. It suggests opportunities for future research involving closed-loop environmental systems, neuroadaptive interfaces, generative design optimization, and human-in-the-loop spatial decision systems, provided that such applications are accompanied by transparent validation and appropriate ethical safeguards.

### 6.5 . Implications for evidence-based neuroarchitecture

Taken together, the findings support a shift from technology accumulation toward evidence architecture. Future progress will depend not only on acquiring more EEG, eye-tracking, autonomic, or immersive data, but on establishing transparent relationships among stimulus definition, exposure conditions, measurement protocols, cognitive-emotional interpretation, and design translation.

The ISCF and EBN-MM address complementary aspects of this problem. The ISCF provides a process-oriented structure for locating evidence within the spatial-stimulus-to-design-translation chain, whereas the EBN-MM evaluates how far individual contributions progress from interpretation toward operationalization. Used together, the frameworks make it possible to distinguish methodological complexity from translational maturity and to identify where additional evidence is required before design recommendations can be justified.

## 7 Limitations

Several limitations should be considered when interpreting the findings. First, the review was restricted to Scopus and Web of Science Core Collection. Although these databases provide broad interdisciplinary coverage, relevant studies indexed exclusively in discipline-specific databases may not have been captured. Second, the expanded search was designed to move beyond the literal terms neuroarchitecture and neuro-architecture by incorporating architectural and spatial cognition, navigation and wayfinding, immersive environments, and neurophysiological or behavioral built-environment research. This broader strategy improved conceptual coverage but also increased boundary ambiguity, requiring explicit scope decisions during screening and final eligibility auditing.

Third, the archived project records preserve the structure, database-specific search sets, and retrieval counts of the expanded search, but the exact line-by-line Boolean syntax was not retained for every search set. These expressions were therefore not retrospectively reconstructed. This limitation is reported explicitly to distinguish verified search documentation from reconstructed information.

Fourth, the final corpus intentionally includes heterogeneous evidence types, including original empirical research, systematic and narrative reviews, conceptual contributions, methodological/framework papers, and one protocol. This breadth is appropriate for systematic mapping and field-level maturity assessment but precludes treating the complete corpus as a homogeneous empirical sample. Denominators were therefore separated according to analytical purpose, particularly for measurement-frequency analyses.

Fifth, study-level coding necessarily involved interpretive decisions when assigning EBN-MM stages and ISCF primary layers. To limit overinterpretation, classifications followed explicit operational rules, uncertain information was not imputed, and Stage IV was defined conservatively. Nevertheless, these classifications should be understood as structured analytical interpretations rather than externally validated psychometric scales.

Sixth, methodological heterogeneity across the retained literature limits direct comparison of physiological metrics, exposure protocols, participant populations, and outcome definitions. The present study therefore reports evidence distributions and methodological patterns rather than pooled effect sizes. Finally, the review period ended in 2025; subsequent publications may alter the distribution of emerging approaches, particularly AI-supported and adaptive neuroarchitectural systems.

## 8 Conclusion

This systematic mapping review examined the development of neuroarchitecture and closely related architectural and spatial-cognition research between 2015 and 2025. Expanded searches of Scopus and Web of Science Core Collection identified 510 records; following duplicate removal, screening, and final eligibility auditing, 121 studies were retained. The resulting evidence base demonstrates rapid recent growth, with 66.9% of retained studies published between 2023 and 2025, but also substantial conceptual and methodological heterogeneity.

The findings show that contemporary neuroarchitecture is not defined by a single experimental technology. Among the 61 human empirical studies, questionnaire/self-report assessment was identified in 63.9%, while behavioral task/performance measures and EEG were each present in 52.5%. Eye tracking, EDA/GSR, HRV, fMRI, and fNIRS contributed additional but less frequently represented evidence streams. This pattern supports an interpretation of neuroarchitecture as an increasingly multimethod field integrating experiential, behavioral, and physiological evidence.

The EBN-MM further demonstrates that field maturity is distributed almost evenly across Conceptual Interpretation (30.6%), Experimental Validation (35.5%), and Evidence Integration (33.1%), whereas Adaptive Design Operationalization remains exceptional (0.8%). Consistently, the ISCF mapping shows the strongest concentration at L3—Neurophysiological Measurement (38.8%), while only 9.1% of studies primarily reached Evidence-Based Design Translation and 0.8% reached Adaptive/AI-Supported Design.

These findings identify the principal challenge for future neuroarchitecture research as one of translation rather than measurement alone. The field has developed substantial capacity to characterize human responses to architectural environments, but comparatively little evidence currently demonstrates how these measurements can be converted into validated design decisions or adaptive spatial systems.

The Immersive Spatial Cognition Framework (ISCF) and Evidence-Based Neuroarchitecture Maturity Model (EBN-MM) provide complementary structures for addressing this gap. The ISCF organizes evidence across the pathway from spatial stimulus and exposure to measurement, interpretation, design translation, and adaptive application, while the EBN-MM distinguishes conceptual development, empirical validation, evidence integration, and operationalization. Future research should prioritize transparent reporting, reproducible multimethod protocols, ecological validation, stronger links between measured responses and architectural variables, and ethically grounded human-in-the-loop design systems. Such developments would support the transition from an expanding interdisciplinary evidence base toward genuinely evidence-based and design-operational neuroarchitecture.

## Data Availability

The original contributions presented in the study are included in the article/Supplementary material, further inquiries can be directed to the corresponding author.

## References

[ref1] Albayrak-Kutlay G. (2025). Exploring VR and neuroscience methodologies in interior design: a systematic review. Hum. Behav. Emerg. Technol. 2025:7410855. doi: 10.1155/hbe2/7410855

[ref2] Attaianese E. Barilà M. Perillo M. (2025). Exploring neuroscientific approaches to architecture: design strategies of the built environment for improving human performance. Buildings 15:3524. doi: 10.3390/buildings15193524

[ref3] Banaei M. Hatami J. Yazdanfar A. Gramann K. (2017). Walking through architectural spaces: the impact of interior forms on human brain dynamics. Front. Hum. Neurosci. 11:477. doi: 10.3389/fnhum.2017.00477 PMC5627023 29033807

[ref4] Bower I. Tucker R. Enticott P. G. (2019). Impact of built environment design on emotion measured via neurophysiological correlates and subjective indicators: a systematic review. J. Environ. Psychol. 66:101344. doi: 10.1016/j.jenvp.2019.101344

[ref5] Browning W. D. Ryan C. O. Clancy J. O. (2014). 14 Patterns of Biophilic Design: Improving Health and Well-Being in the Built Environment. New York: Terrapin Bright Green.

[ref7] Chatterjee A. Vartanian O. (2014). Neuroaesthetics. Trends Cogn. Sci. 18, 370–375. doi: 10.1016/j.tics.2014.03.003, 24768244

[ref8] Coburn A. Vartanian O. Chatterjee A. (2017). Buildings, beauty, and the brain: a neuroscience of architectural experience. J. Cogn. Neurosci. 29, 1521–1531. doi: 10.1162/jocn_a_01146, 28493809

[ref10] de Paiva A. (2025). Methodological challenges in neuroarchitecture: towards a better understanding on how architecture affects cognitive reserve. Intell. Build. Int. 17, 4–12. doi: 10.1080/17508975.2025.2493696

[ref11] de Paiva A. Jedon R. (2019). Short- and long-term effects of architecture on the brain: toward theoretical formalization. Front. Archit. Res. 8, 564–571. doi: 10.1016/j.foar.2019.07.004

[ref13] Eberhard J. P. (2009). Applying neuroscience to architecture. Neuron 62, 753–756. doi: 10.1016/j.neuron.2009.06.001, 19555644

[ref14] Edelstein E. A. Macagno E. (2012). “Form follows function: bridging neuroscience and architecture,” in Sustainable Environmental Design in Architecture: Impacts on Health, (Springer), 27–41. doi: 10.1007/978-1-4419-0745-5_3

[ref16] Gramann K. Fairclough S. H. Zander T. O. Ayaz H. (2017). Editorial: trends in Neuroergonomics. Front. Hum. Neurosci. 11:165. doi: 10.3389/fnhum.2017.00165 PMC5380744 28424601

[ref17] Grobman Y. J. (2026). Bridging space perception, emotions, and artificial intelligence in neuroarchitecture. Brain Sci. 16:131. doi: 10.3390/brainsci16020131, 41750132 PMC12938172

[ref19] Higuera-Trujillo J. L. Llinares C. Macagno E. (2021). The cognitive-emotional design and study of architectural space: a scoping review of neuroarchitecture and its precursor approaches. Sensors 21:2193. doi: 10.3390/s21062193, 33801037 PMC8004070

[ref21] Kalantari S. Neo J. R. J. (2020). Virtual environments for design research: lessons learned from use of fully immersive virtual reality in interior design research. J. Inter. Des. 45, 27–42. doi: 10.1111/joid.12171

[ref22] Kim N. Gero J. S. (2023). “Neurophysiological responses to biophilic design: a pilot experiment using VR and EEG,” in Design Computing and Cognition ‘22, ed. Gero J. S. (Cham: Springer), 235–253.

[ref24] Kuliga S. F. Thrash T. Dalton R. C. Hölscher C. (2015). Virtual reality as an empirical research tool—exploring user experience in a real building and a corresponding virtual model. Comput. Environ. Urban. Syst. 54, 363–375. doi: 10.1016/j.compenvurbsys.2015.09.006

[ref27] Llinares C. Higuera-Trujillo J. L. Serra J. (2021). Cold and warm coloured classrooms: effects on students’ attention and memory measured through neurophysiological responses. Build. Environ. 196:107726. doi: 10.1016/j.buildenv.2021.107726

[ref29] Mallgrave H. F. (2013). Architecture and Embodiment: The Implications of the New Sciences and Humanities for Design. New York: Routledge.

[ref30] Medhat Assem H. Mohamed Khodeir L. Fathy F. (2023). Designing for human wellbeing: the integration of neuroarchitecture in design—a systematic review. Ain Shams Eng. J. 14:102102. doi: 10.1016/j.asej.2022.102102

[ref9001] Ouzzani M. Hammady H. Fedorowicz Z. Elmagarmid A. (2016). Rayyan—a web and mobile app for systematic reviews. Syst. Rev. 5:210. doi: 10.1186/s13643-016-0384-4 PMC5139140 27919275

[ref31] Page M. J. McKenzie J. E. Bossuyt P. M. Boutron I. Hoffmann T. C. Mulrow C. D. . (2021). The PRISMA 2020 statement: an updated guideline for reporting systematic reviews. BMJ 372:n71. doi: 10.1136/bmj.n71, 33782057 PMC8005924

[ref32] Rizzo A. Koenig S. T. Parsons T. D. (2023). Virtual reality applications for assessment and treatment in psychiatry. Curr. Psychiatry Rep. 25, 1–11.

[ref33] Shemesh A. Talmon R. Karp O. Amir I. Bar M. Grobman Y. J. (2017). Affective response to architecture—investigating human reaction to spaces with different geometry. Archit. Sci. Rev. 60, 116–125. doi: 10.1080/00038628.2016.1266597

[ref35] Sternberg E. M. Wilson M. A. (2006). Neuroscience and architecture: seeking common ground. Cell 127, 239–242. doi: 10.1016/j.cell.2006.10.012, 17055420

[ref36] Taherysayah F. Malathouni C. Liang H.-N. Westermann C. (2024). Virtual reality and electroencephalography in architectural design: a systematic review of empirical studies. J. Build. Eng. 85:108611. doi: 10.1016/j.jobe.2024.108611

[ref37] Ulrich R. S. Zimring C. Zhu X. DuBose J. Seo H. Choi Y. . (2008). A review of the research literature on evidence-based healthcare design. HERD 1, 61–125. doi: 10.1177/193758670800100306, 21161908

[ref38] Valentine C. (2023). Health implications of virtual architecture: an interdisciplinary exploration of the transferability of findings from Neuroarchitecture. Int. J. Environ. Res. Public Health 20:2735. doi: 10.3390/ijerph20032735 PMC9915076 36768106

[ref39] Vartanian O. Navarrete G. Chatterjee A. Fich L. B. Leder H. Modrono C. . (2013). Impact of contour on aesthetic judgments and approach-avoidance decisions in architecture. Proc. Natl. Acad. Sci. USA 110, 10446–10453. doi: 10.1073/pnas.1301227110, 23754408 PMC3690611

[ref40] Yin J. Zhu S. MacNaughton P. Allen J. G. Spengler J. D. (2018). Physiological and cognitive performance of exposure to biophilic indoor environment. Build. Environ. 132, 255–262. doi: 10.1016/j.buildenv.2018.01.006

