# Supplementary material for: Toward evidence-based neuroarchitecture: a systematic mapping and framework for immersive spatial cognition
Source: Front Psychol. 2026 Sep 7;17:1919278. doi: 10.3389/fpsyg.2026.1919278 (PMC13590291; doi:10.3389/fpsyg.2026.1919278)
Supplement: Supplementary file 1 [file Supplementary_file_1.docx]

Supplementary Material S1
Expanded Search Strategy and Database Retrieval Protocol

*Systematic Mapping Review of Neuroarchitecture and Architectural/Spatial Cognition, 2015–2025*

# S1.1 Purpose

This supplementary document records the final expanded database-search architecture used to construct the review corpus. It reports the databases, search fields, temporal and document-type restrictions, conceptual search domains, auditable search-set identifiers, retrieval counts, deduplication totals, and export procedure. The final retrieval counts are synchronized with the Frozen Master Dataset v9 and the final PRISMA audit.

# S1.2 Databases and Search Fields

| Database | Search field | Coverage |
| --- | --- | --- |
| Scopus | TITLE-ABS-KEY | Title, abstract, and author keywords |
| Web of Science Core Collection | Topic (TS) | Title, abstract, author keywords, and Keywords Plus |

# S1.3 Eligibility Restrictions Applied to Searches/Screening

- Publication period: 2015–2025.
- Language: English.
- Document type: peer-reviewed journal Article and Review.
- Conference proceedings, editorials, notes, book chapters, letters, dissertations, and other non-peer-reviewed publication types were excluded.
- For publications appearing online in 2025 but assigned to a 2026 issue, eligibility was based on first online/version-of-record publication by 31 December 2025; this temporal rule is documented in S6C.

# S1.4 Expanded Conceptual Search Domains

Following reviewer concern that a search restricted to the literal terms “neuroarchitecture” and “neuro-architecture” was too narrow for the manuscript’s broader claims, the search was expanded using multiple complementary search sets. The expanded strategy targeted the following concept families:

- Neuroarchitecture / neuro-architecture and closely related architectural-neuroscience terminology.
- Architectural cognition, spatial cognition, environmental cognition, cognitive mapping, spatial memory, spatial perception, wayfinding, and navigation.
- Built-environment and architectural-space research using EEG, fMRI/fNIRS, eye tracking, HRV/ECG, EDA/GSR, heart rate, behavioral tasks, or structured self-report.
- Virtual reality, immersive environments, CAVE/projection environments, and other digitally simulated architectural/spatial exposures.
- Restorative, biophilic, healthcare, learning, workplace, residential, and urban settings only when the built environment and cognitive/neurophysiological response were central to the study.

# S1.5 Audited Search-Set Retrieval Counts

| Database | Search set | Records | Database subtotal |
| --- | --- | --- | --- |
| Scopus | S1 | 221 | 235 |
| Scopus | S2 | 4 | 235 |
| Scopus | S3 | 10 | 235 |
| Web of Science | W1 | 220 | 275 |
| Web of Science | W2 | 10 | 275 |
| Web of Science | W3 | 45 | 275 |

Across all six expanded search sets, 510 records were retrieved (Scopus n = 235; Web of Science n = 275). After deduplication, 304 unique records remained for screening. The PRISMA duplicate-record count is therefore 206 (510 − 304). Rayyan’s potential-duplicate-group count must not be reported as the number of duplicate records removed.

# S1.6 Search Syntax Transparency Note

The archived project workbooks preserve the expanded search-set identifiers (S1–S3 and W1–W3), database subtotals, and final retrieval counts, but they do not preserve the exact line-by-line Boolean syntax for every expanded set. Accordingly, no exact Boolean string has been reconstructed from memory in this supplementary document. This is a deliberate traceability safeguard. If the original Scopus and Web of Science search-history exports remain available, their exact query syntax should be appended to the submission archive without changing the audited retrieval counts reported here.

# S1.7 Export, Deduplication, and Screening

All search-set results were exported in bibliographic format and combined in Rayyan. Duplicate removal was audited arithmetically from the retrieval totals: 510 identified records − 304 unique screened records = 206 duplicate records removed. The final Rayyan screening set contained 304 unique records. Following screening and quality-control review, 125 records entered the final eligibility/data-extraction audit. Four additional boundary/date-range exclusions were applied (S068, S113, S117, and S125), producing the frozen corpus of 121 retained studies.

# S1.8 Reproducibility and Scope Statement

The expanded search was designed to map neuroarchitecture together with directly related architectural/spatial-cognition research rather than only publications self-labeled with the term neuroarchitecture. The final scope therefore includes empirical, review, conceptual, methodological, and protocol evidence when architecture/built environment and cognition, behavior, or neurophysiological response are central. The complete retained corpus is provided in S4 and S5; the coding rules are documented in S6A–S6C; and S7 provides the final analytical dataset.

**Finalized against Frozen Master Dataset v9 — 7 August 2026**
