# Supplementary material for: Toward evidence-based neuroarchitecture: a systematic mapping and framework for immersive spatial cognition
Source: Front Psychol. 2026 Sep 7;17:1919278. doi: 10.3389/fpsyg.2026.1919278 (PMC13590291; doi:10.3389/fpsyg.2026.1919278)
Supplement: Supplementary file 2 [file Supplementary_file_2.docx]

Supplementary Material S6A
Coding Manual for the Analytical Synthesis of Neuroarchitecture Studies

*Systematic Mapping Review, 2015–2025
Final version aligned with Frozen Master Dataset v9 and S7 Final Analytical Dataset*

# 1. Purpose

This manual defines the standardized coding procedures used to transform the frozen study-level Master Dataset into the final Analytical Dataset. It establishes the evidence threshold, controlled vocabulary, operational rules, binary coding logic, and audit procedures used for exposure technologies, measurement modalities, physiological metrics, architectural variables and environmental contexts, cognitive and emotional constructs, and behavioral outcomes. It is used together with S6B (Controlled Vocabulary), S6C (Coding Decision Log), and S7 (Final Analytical Dataset).

# 2. Scope and Unit of Analysis

The unit of analysis is one publication. Each retained publication constitutes one analytical case and retains its unique Study_ID across the Master Dataset, S6C, S7, tables, figures, and manuscript text. Multiple coding is permitted when a publication explicitly reports more than one applicable exposure, modality, metric, architectural variable, construct, or behavioral outcome.

# 3. Evidence Threshold

Codes are assigned only when the relevant information is explicitly reported in the title, abstract, keywords, or verified full text. Inference-based coding is prohibited. A physiological metric is not inferred from a measurement modality alone; a cognitive or emotional construct is not inferred from a physiological signal alone; and a specific architectural variable is not inferred from a generic environmental context. Missing or unspecified information remains uncoded rather than imputed.

# 4. Analytical Domains

Six principal analytical domains are used: (1) exposure technologies; (2) measurement modalities; (3) physiological metrics; (4) architectural variables and environmental contexts; (5) cognitive and emotional constructs; and (6) behavioral outcomes. Exposure technologies are separated from measurement modalities: for example, immersive VR defines how an environment is presented, whereas EEG, HRV, EDA/GSR, eye tracking, fMRI, and fNIRS define how responses are measured.

# 5. General Coding Rules

1. One publication constitutes one analytical case.
2. Multiple coding is allowed when explicitly supported by the source.
3. Only explicitly reported information may be coded; inference-based coding is prohibited.
4. Exposure/presentation technologies and measurement modalities are independent dimensions.
5. Physiological signals and derived metrics are coded separately from cognitive/emotional constructs.
6. Behavioral outcomes are coded independently from physiological measurements.
7. Review-level mentions are excluded from original empirical modality and metric frequency counts.
8. Missing or unspecified details remain uncoded rather than imputed.
9. Every positive analytical code must remain traceable to Study_ID and supporting evidence.
10. Method-focused empirical studies are distinguished from direct architectural-effect evidence.
11. Non-human empirical studies are retained as a separate evidence stratum and excluded from human denominators.
12. Protocol/planned measurements are retained for methodological synthesis but are not counted as observed empirical measurements.

# 6. Binary Coding Logic

Where S7 analytical matrices use binary coding, 1 = explicitly reported/present and 0 = not reported under the defined evidence threshold. A value of 0 therefore means 'not coded/reported'; it is not proof that the characteristic was absent from the underlying experiment. Multiple values of 1 may occur within the same study.

# 7. Publication-Type and Denominator Rules

| Evidence stratum | Retained in S7 | Use in empirical frequencies |
| --- | --- | --- |
| Human empirical | Yes | Yes, for applicable empirical analyses |
| Method-focused empirical | Yes | Yes for method counts; distinguish from direct design-effect evidence |
| Non-human empirical | Yes | No for human participant/modality denominators |
| Review / conceptual / framework | Yes | No for original empirical modality/metric frequencies |
| Protocol / planned methods | Yes | No for observed empirical frequencies |
| Excluded records | No | No |

# 8. Measurement and Metric Rules

- EEG is coded as a measurement modality only when original EEG acquisition is reported. Theta, alpha, beta, gamma, ERP/VEP components, band ratios, and derived indices are coded only when explicitly reported.
- Eye tracking is coded separately from its derived metrics. Fixation count/time, saccades, gaze velocity/trajectory, heat maps, AOI measures, and pupillometry are not inferred from the modality alone.
- HRV/ECG, heart rate, and EDA/GSR are treated as distinct measurement families. LF, HF, LF/HF, RMSSD, SDNN, and phasic/SCR measures require explicit source support.
- fMRI metrics include source-supported BOLD/region activation and, when reported, multivoxel/RSA/searchlight analyses. Review-level fMRI mentions are not original observations.
- fNIRS is coded only when the source explicitly identifies functional near-infrared spectroscopy. Generic cerebral hemoglobin density/concentration is not relabeled as fNIRS without explicit methodological support.

# 9. Analytical Boundary Decisions

Exceptional eligibility, temporal, population, measurement, and methodological decisions are documented in S6C. The final audit includes, among others, separation of review-level measurements from empirical measurements; treatment of S061 as non-human evidence; treatment of S085 as methodological/protocol evidence; correction of S027 to behavioral/self-report measurement only; and removal of an unsupported fNIRS label from S051.

# 10. Quality Assurance and Traceability

- The Frozen Master Dataset v9 is the single source of truth for final analytical outputs.
- S7 Final is derived only from the frozen master and contains 121 retained records; four excluded records are not included in S7.
- Study_ID is preserved across all supplementary files and manuscript outputs.
- Final cross-validation checks measurement modality against named metric families.
- Review/conceptual and protocol-level mentions are analytically restricted before frequency calculations.
- Unsupported exact metrics remain at the source-reported level of granularity rather than being inferred.

# 11. Frequency and Cross-Domain Analysis

A study using multiple exposure technologies or measurement modalities contributes once to each applicable category. Percentages in multi-response tables may therefore sum to more than 100%. Physiological metrics, constructs, and behavioral outcomes are summarized separately. Cross-domain analyses may examine exposure–measurement pairings, modality–metric relationships, construct distributions across architectural contexts, and multimodal combinations. Denominators must follow the publication-type rules in Section 7.

# 12. Final Supplementary File Relationships

S5 contains the frozen study-level Master Dataset. S6A defines the coding rules. S6B provides the controlled vocabulary. S6C records exceptional/boundary decisions. S7 is the final analytical dataset derived from S5. Together these files provide the audit trail linking the retained evidence corpus to all tables, figures, Results, and framework reconstruction.

**Freeze date: 7 August 2026**
